# ﻿Contributions to Ecuadorian butterworts (Lentibulariaceae, *Pinguicula*): two new species and a re-evaluation of *Pinguiculacalyptrata*

**DOI:** 10.3897/phytokeys.222.98139

**Published:** 2023-03-24

**Authors:** Álvaro J. Pérez, Francisco Tobar, Kevin S. Burgess, Tilo Henning

**Affiliations:** 1 Herbario QCA, Escuela de Ciencias Biológicas, Pontificia Universidad Católica del Ecuador, Av. 12 de Octubre 1076 y Roca, Apartado 17-01-2184, Quito, Ecuador Pontificia Universidad Católica del Ecuador Quito Ecuador; 2 Área de Investigación y Monitoreo de Avifauna, Aves y Conservación-BirdLife en Ecuador, Quito, Ecuador Área de Investigación y Monitoreo de Avifauna Quito Ecuador; 3 Herbario Nacional del Ecuador, Instituto Nacional de Biodiversidad, Pasaje Rumipamba 341 y Av. de los Shyris, 170135, Quito, Pichincha, Ecuador Herbario Nacional del Ecuador, Instituto Nacional de Biodiversidad Quito Ecuador; 4 Department of Biology, College of Letters & Sciences, Columbus State University, University System of Georgia, 4225 University Ave, Columbus, GA 31907, USA Columbus State University Columbus United States of America; 5 Leibniz Centre for Agricultural Landscape Research (ZALF), Eberswalder Str. 84, 15374, Müncheberg, Germany Leibniz Centre for Agricultural Landscape Research Müncheberg Germany

**Keywords:** Amotape-Huancabamba, Andes, Cordillera del Cóndor, distribution, Ecuador, endemism, Jimbura, morphological variation, new species, taxonomy

## Abstract

Comparatively few species of the insectivorous genus *Pinguicula* L. have been recognized in South America so far. In recent years, a number of narrowly endemic taxa from the Andes have been described that simultaneously refined the broad taxonomic concepts of the “historical” species. Here, we describe two striking new species from Southern Ecuador that further condense the circumscription of *Pinguiculacalyptrata* Kunth. *Pinguiculajimburensis***sp. nov.** and *P.ombrophila***sp. nov.** are clearly beyond the taxonomic scope of the known species and consequently described as new to science. The deviating morphological features of the two new taxa are described and illustrated and the remaining morphological spectrum of *P.calyptrata* in Ecuador is outlined. The two new species add to the exceptional biodiversity in the Amotape-Huancabamba Zone and underline its importance as a biodiversity hotspot in urgent need of protection.

## Abstract (es)

Actualmente pocas especies del género insectívoro *Pinguicula* L. se reconocen de forma comparativa en América del Sur. En los últimos años, se han descrito varios taxones micro-endémicos de los Andes que simultáneamente han refinado los amplios conceptos taxonómicos de las especies “históricas”. Aquí, describimos dos nuevas especies sorprendentes del sur del Ecuador que condensan aún más la circunscripción de *Pinguiculacalyptrata* Kunth. *Pinguiculajimburensis***sp. nov.** and *P.ombrophila***sp. nov.** están más allá del alcance taxonómico de las especies conocidas y, en consecuencia, descritas como nuevas para la ciencia. Se describen e ilustran los caracteres morfológicos divergentes de los dos nuevos taxones y se discute la variación morfológica restante de *P.calyptrata* en Ecuador. Las dos nuevas especies se suman a la excepcional biodiversidad de la zona Amotape-Huancabamba y se realza su importancia como un punto crítico de biodiversidad que necesita protección urgente.

## ﻿Introduction

*Pinguicula* L., the second largest genus of the most specious family of carnivorous plants – Lentibulariaceae (Lamiales) – comprises approximately 115 species (Fleischmann 2021). The genus is distributed throughout Eurasia and the Americas, is largely absent from Africa (only Morocco) and not present in Oceania and Antarctica. Roughly half of all taxa occur in Latin America with a clear center of diversity in Mexico, with about 40 endemic species alone. Despite the high diversity in Central America, Mexico and the Caribbean, only a few species are known from South America and restricted to the Andean mountain chain. At the moment, most authors accept seven species here, namely: *P.antarctica* Vahl, *P.calyptrata* Kunth, *P.elongata* Benj., *P.involuta* Ruiz & Pav., *P.jarmilae* Halda & Malina, *P.nahuelbutensis* Gluch and *P.rosmarieae* Casper, Bussmann & T.Henning. All species belong to the monophyletic sect. Ampullipalatum Casper within the subgenus Isoloba, with the exception of *P.elongata* (sect. Heterophylliformis (Casper) Fleischmann & Roccia within subgen. Themnoceras).

Significantly, all recently described species are narrow endemics (*P.jarmilae*, *P.nahuelbutensis* & *P.rosmarieae*), whereas the earlier described taxa usually have larger ranges. This already indicates that the relatively low number of taxa known from (Andean) South America might be, at least partially, rather a result of under-collection and a lack of taxonomic studies, than true species poverty.

In Ecuador, the genus is to this date only represented by *Pinguiculacalyptrata* Kunth (Taylor 1975; Jørgensen and León-Yánez 1999). The holotype for this species was collected in the páramo of Saraguro (Loja Province, Ecuador) by Alexander von Humboldt and Aimé Bonpland during their five years expedition to South America (1799–1804). It was published in Karl Sigismund Kunth’s “Nova Genera et Species Plantarum” (Humboldt et al. 1818) with a brief and superficial description and without an illustration as was customary at the time. The only taxonomic study that subsequently dealt with this group of plants in Ecuador dates back 48 years (Taylor 1975). His treatment of the Lentibulariaceae for the Flora of Ecuador was, however, mostly based on the scarce herbarium material available at the time and could not deliver a critical review of the genus for Ecuador.

### ﻿*Pinguicula* in the Amotape-Huancabamba Zone and adjacent Andean regions

The genus *Pinguicula* is particularly species-rich in Latin America. However, the taxonomic richness south of the genus´ center of diversity: Mexico, Central America and the Caribbean, is limited, and most of the few taxa recognized are reported to have large latitudinal distributions. Lately, the continuous botanical exploration of the Andes and especially of the so-called Amotape-Huancabamba Zone (hereafter AHZ), however, shows a more complex picture for the genus in this area than hitherto believed. This phytogeographical zone has been revealed as an area characterized by an exceptional biodiversity and a noticeable accumulation of narrowly endemic taxa (Weigend 2002, 2004). This has been demonstrated for many groups of organisms including numerous angiosperm taxa (e.g. Deanna et al. 2018; Escobar et al. 2018; Acuña-Castillo et al. 2021; Landis et al. 2021;Tejedor and Calatayud 2022; Vieu et al. 2022) and only very recently also for the Lentibulariaceae. With the description of *P.rosmarieae*, the first narrowly endemic *Pinguicula* has been documented for the AHZ (Casper et al. 2020). And the recent description of *Utriculariaamotape-huancabambensis* (Henning et al. 2021) revealed the same narrow endemism for the other large genus in the family.

However, despite constantly growing botanical research activities and new collections, taxon delimitation in *Pinguicula* remains problematic. This is particularly true for *Pinguiculacalyptrata*, which is distributed from Central Colombia, throughout Ecuador and most of the AHZ until the North Peruvian Departamentos Cajamarca, Amazonas and San Martín (all specimens from Bolivia that we are aware of are misidentified *P.involuta*) and is unsatisfactorily defined in its entirety (Casper et al. 2020). Caspers concept of *Pinguicula*, in both systematics and alpha-taxonomy, was very flower focused (Fleischmann 2021). Species delimitation and the infrageneric classification of the genus in his works largely founded on morphological character complexes of the corolla, traditionally thought to be evolutionary stable. Instead, they were often subject to rapid diversifications and thereby prone to misinterpretations as recent molecular studies repeatedly revealed (Lustofin et al. 2020; Shimai et al. 2021, see Fleischmann 2021 for a detailed discussion). In the course of studying the Andean taxa, the late Siegfried Jost Casper (1929–2021) also based his allocation of new collections/observations on the gross flower morphology and (for the time being) tended to classify any observation from the Northern Andes with more or less spreading rosettes and bluish flowers with a yellow palate as *P.calyptrata* (pers. correspondence). However, similar to the situation reported for a number of Cuban species (Domínguez et al. 2013), gradual morphological variation is found between most of the populations, varying with latitudinal and longitudinal distribution and elevation. Furthermore, on site-conditions such as e.g. moisture, soil type, associated vegetation and presumably the local pollinator fauna – a commonly ignored aspect in the context of speciation in the mosaic-like microhabitats of the Andes – affect genetic exchange between populations and facilitate diversification (Kay and Sargent 2009; Van der Niet et al. 2014). These dynamic and diverse networks of relationships and interactions can have a catalyzing effect on such character complexes that can be subject to rapid alteration and adaptation. In particular the plasticity of e.g. flower size, shape and color is greater than traditionally assumed and divergent versus convergent developments can be difficult to determine, potentially complicating an unambiguous assignment of taxa (Fleischmann 2021). With the ongoing botanical exploration of the (remaining) remote paramo habitats of Ecuador and Peru (and other Andean countries), the real extent of the morphological spectrum becomes increasingly recognizable and, in the case of *P.calyptrata*, quickly goes beyond the narrow scope of the protologue.

Besides biogeographical data and habitat preferences, the overall bauplan of *Pinguicula* provides only a limited number of characters to take into consideration for taxon delimitation. Basically, the plant body in this genus is reduced to leaves and flowers and morphological characterization boils down to a handful of characters delivered by these two organs. In the light of the aforementioned gradual variability between populations and the known uncertainties in species recognition in Andean South America (e.g. *P.huilensis* – Cuatrecasas, 1945; *P.chilensis* and *P.antarctica* – Gluch 2017; Roccia 2018) it appears appropriate that species delimitation must be subject to significant, quantifiable and consistent morphological differences (see stalk length debate in Gluch 2017 and Roccia 2018) and, in the case of the AHZ, must be considered in relation to omnipresent and often sympatric “typical” *P.calyptrata*. Nevertheless, lumping all taxa of *Pinguicula* from the Northern Andes together under *P.calyptrata* due to a superficial similarity in their flower and rosette morphology would not do the actual diversity any justice and could leave a false impression regarding the actual threat status of potential narrow endemic taxa.

Taking these considerations into account, most Ecuadorian populations of *Pinguicula* fall into the range of *P.calyptrata* and this species in its (original) unsharp definition is present in many suitable Andean habitats sampled, although a more fine-grade analysis of *P.calyptrata* s.l., applying population genetics, is urgently needed and strongly encouraged. However, in two localities, two distinct taxa could be found growing sympatrically. The collections from the Cerro Plateado Biological Reserve show two taxa, that clearly differ in leaf morphology and rosette shape, albeit having similar flowers. Conversely, collections from Yacuri National Park show two taxa, differing considerably in both character complexes: leaves/rosettes and detailed flower morphology.

Despite the disparate grade of morphological dissimilarity observed for the two taxon pairs, the new taxa are both separated at species level to meet the needs of a conservation assessment under the IUCN criteria (IUCN Standards and Petitions Committee 2022) and facilitate potential protective measures. Whether a different taxonomic concept might ultimately be more appropriate to reflect the close relationship among (some) Andean *Pinguicula* species remains open to future studies, ideally within the framework of a more comprehensive approach using both phylogenetic and population genetic methods.

The new taxa are morphologically clearly distinguishable and a consistent spatial segregation is evident. Both new taxa share their habitat with rather typical *P.calyptrata* and are ecologically separated by contrasting habitat requirements, a pattern reported already for *P.rosmarieae* and *P.calyptrata* from North Peru (Casper et al. 2020). Similarly, the taxon pairs found in Ecuador also grow in close proximity to each other without any direct spatial overlap. No morphologically intermediate plants were found, indicating that the taxa here described are discrete reproductive units without naturally occurring hybrid individuals (unlike recently reported for *Utricularia* – Henning et al. 2021).

Consequently, *P.jimburensis* sp. nov. and *P.ombrophila* sp. nov. are described as species new to science. All aspects of their biology are presented and discussed. Furthermore, the morphological spectrum of *P.calyptrata* throughout the known Ecuadorian populations is outlined and illustrated. A key to the Ecuadorian species is provided and an up-to-date assessment of the taxonomic situation of *Pinguicula* in the Northern Andes is made.

## ﻿Materials and methods

In the current study, we document two new narrow endemic *Pinguicula* species from southern Ecuador as a result of recent botanical explorations. We conduct detailed morphological analyses for these new species, provide a taxonomic description, present images for all floral and vegetative plant structures, and provide a distribution map. The conservation status of the newly described species and their relationships with other species are also discussed.

Furthermore, the morphological spectrum of *P.calyptrata* throughout the known Ecuadorian populations is outlined and illustrated. The populations of this species have been collected from north to south in all the Ecuadorian Andean provinces from 2100 to 4100 m.

We consulted pertinent literature, examined specimens at ECUAMZ, GUAY, LOJA, QCA and QCNE herbaria (Thiers 2023), and high-resolution images of type material for Neotropical taxa (Tropicos database, https://www.tropicos.org/ and the JSTOR Global Plants website http://plants.jstor.org).

## ﻿Results

### ﻿Key to the Ecuadorian species of *Pinguicula*

**Table:** 

1	leaves (semi) upright, elongated, margins shallowly irregular lobed, flowers without a yellow palate, spur evenly bent (carnassial tooth-like) and with a tapered apex	***Pinguiculajimburensis* sp. nov.**
–	rosettes flat on the ground, leaf margins entire, flowers with a distinct yellow palate, spur angled from the tube, often thickened at the end and with a stubby apex	**2**
2	terrestrial plant, rosette star-like, leaf blades ovate, margins distinctly curled up, flower scapes (much) longer than rosette diameter	***Pinguiculacalyptrata***
–	lithophytic plant, rosette not star-like, leaf blades oblong-obovate-ovate, margins not or only very inconspicuously curled up, flower scapes half as long as rosette diameter	***Pinguiculaombrophila* sp. nov.**

### ﻿Taxonomic treatment

#### ﻿ Pinguicula jimburensis

Taxon classificationPlantaeLamialesLentibulariaceae

Á.J.Pérez, Tobar & T.Henning sp. nov.

C684DE11-7953-54FD-817A-B22640146935

urn:lsid:ipni.org:names:77316266-1

Fig. 1

##### Type.

Ecuador, Loja, Cantón Espíndola, Parroquia Jimbura, Parque Nacional Yacuri, Lagunas Negras de Jimbura, 04°42'46"S, 79°25'50"W, 3400 m, 9 Oct 2022, *Á.J. Pérez et al. 11891* (holotype QCA (fl, fr, spirit collection) barcode: 245581; isotype LOJA (fl) barcode: 43489).

##### Diagnosis.

*Pinguiculajimburensis* belongs to Pinguiculasect.Ampullipalatum and is closely allied to the other North Andean species of the section (*P.calyptrata*, *P.ombrophila* and *P.rosmarieae*). With the latter two it shares the lack of involute leaf margins, but clearly differs from them in its terrestrial habit (vs. litho-/epiphytic) and morphologically by the oblong leaves (vs. widely ovate to rounded). The flowers of *P.jimburensis* lack a distinct yellow palate, a character shared with *P.involuta* and the Peruvian endemic *P.rosmarieae*, but the evenly bent, tooth-like spur is unique among related species. First and foremost, *P.jimburensis* is characterized by erect leaves that are shallow and irregularly lobed. This character is, despite a terminological similarity with the leaves of only distantly related *P.elongata*, unique among all South American taxa.

**Figure 1.**
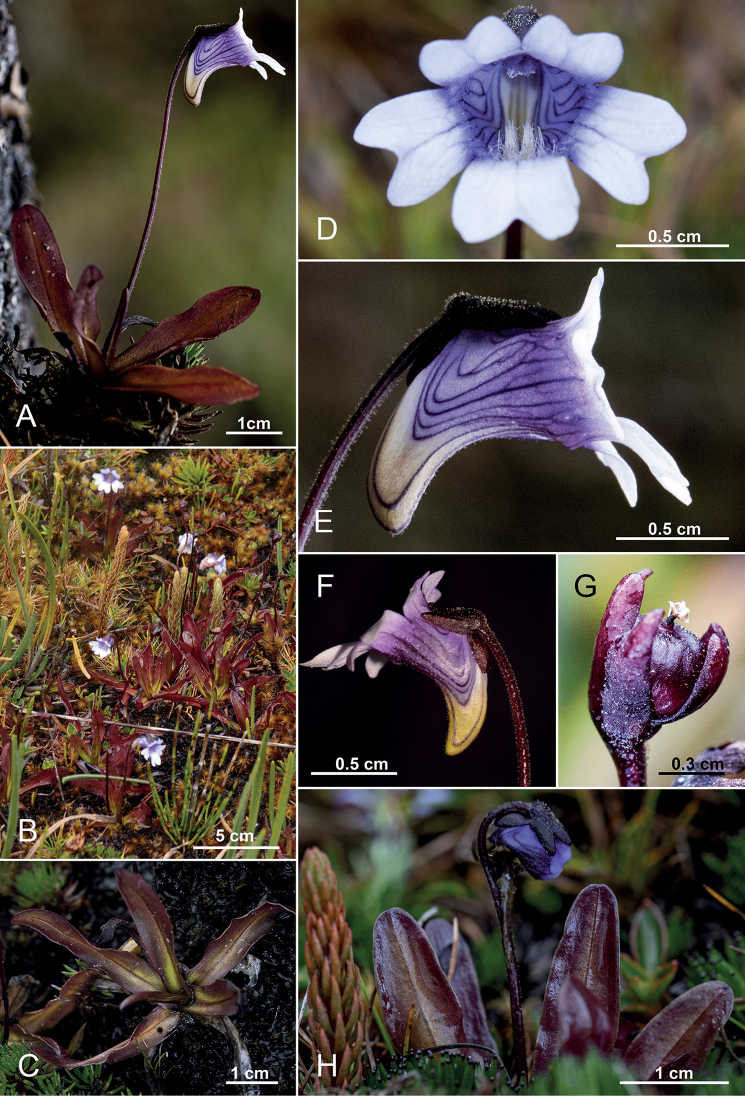
*Pinguiculajimburensis***A** flowering plant of in lateral view **B** stands of *P.jimburensis* at the Lagunas Negras de Jimbura **C** upper view of the rosette **D** flower, frontal view **E** ditto, lateral view **F** ditto, with yellow spur **G** young capsule **H** plant with developing flower bud. (**A–H** from *Á.J. Pérez et al. 11891*). Photos by Kabir Montesinos.

##### Description.

Terrestrial, perennial rosette leaved herb with 1 (–2) flowering scapes. Rhizome ~5 mm long, with numerous fibrous roots 1.5–6 cm long. Leaves (4–) 5–7, erect from the ground, ± succulent, red and fussed with green along the medvein, drying dark purple, (15–) 18–52 (–60) mm long × (2–) 3–8 (–9) mm width, the blades oblong, rounded at the tip, slightly attenuated to the base into a enveloping petiole, the margins are irregularly shallowly lobed, upper surface of lamina covered with stalked glandular hairs. Hibernacula (winter buds, dormant buds) absent. Scapes 1–2 (–3), erect, (30–) 40–60 (–85) mm tall, terete, filiform (0.5–1 mm thick), one-flowered, red, scattered with stalked glandular hairs. Flowers small, 10–12 (–15) mm long (including tube-spur-complex). Calyx two-lipped, red, upper surface of sepals scattered with stalked glandular hairs; upper lip divided into three nearly equal-sized oblong lobes, at apex pointed; lower lip up to ¼ divided into two lobes, but appearing to be entire. Corolla two-lipped, purple-whitish with white lobes; upper lip two-lobed, lobes obovate, ~4–6 mm long and ~3–4.2 mm wide, shallowly notched at the apex; lower lip larger and longer than the upper lip, with three obovate-oblong lobes (the median lobe somewhat larger than the two lateral ones), ~4.5–6 mm wide, each distinctly (up to 1/3 of its length) notched. Tube (tube-spur-complex) at the throat funnel-shaped, on both sides broader than the spur, on the back side higher than the spur, proximally cylindrical (nearly as long as wide), on the ventral side merging without any sharp angle into the cylindrical to cone-like stubby, carnassial tooth-like, at apex tapered white to yellow spur, ~6 mm long; the tube-spur-complex externally whitish -blue to purple lengthwise-striped by parallel veins. Palate simple, weakly developed (not clapper-like), inserted immediately behind (~1–2 mm) the corollas´ lower-lip middle lobe, blue, set with short-stalked glandular hairs, proximally elongated into a short ventral hair strip; each of the two lateral corolla lobes with short-stalked glandular hairs, stretching proximally along on each side of the inner tube wall. Stamens 2, filaments 1.2–2 mm long, anthers dorsifixed, 1 mm, oval, transverse dehiscing. Ovary 1.2 mm, rounded, slightly covered with short-stalked glandular hairs to glabrescent, style 0.5 mm long, stigma 0.5 mm long, campanulate, glabrous. Capsule 3–4 mm, rounded, slightly covered with short-stalked glandular hairs to glabrescent, splitting in 2 valves. Seed numerous, alveolate, ellipsoid, 0.5–0.8 mm long, yellow. Chromosome number unknown.

##### Etymology.

The specific epithet refers to the type locality, Lagunas Negras de Jimbura, which is part of the Yacuri National Park in the Ecuadorian provinces of Loja and Zamora-Chinchipe.

##### Distribution, habitat and associated vegetation.

**Figure 2.**
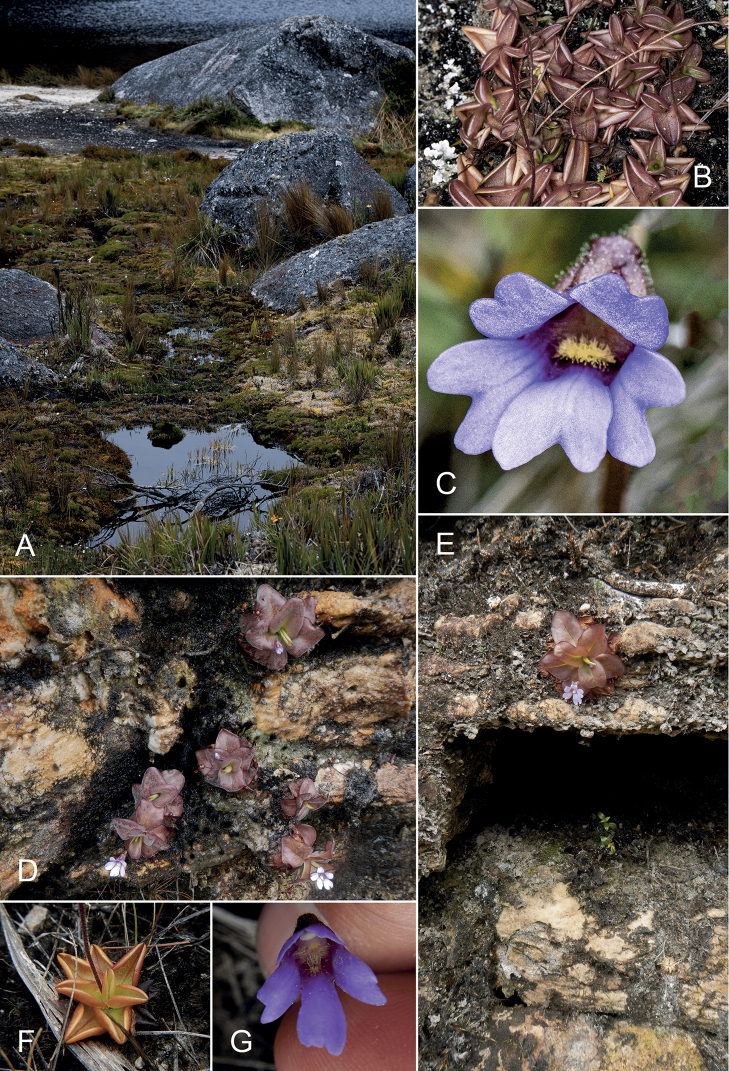
Habitats of the new species and associated *P.calyptrata***A–C** Lagunas Negras de Jimbura **A** swampy areas between rocks next to the Lagoons where *P.jimburensis* is found **B** neighboring stand of *P.calyptrata***C** flower of *P.calyptrata*, frontal view **D–G** Reserva Biológica Cerro Plateado **D** small stand of *P.ombrophila* growing on a vertical rock face **E** single plant on top of a rock overhang **F** rosette of sympatric *P.calyptrata***G** ditto, flower in frontal view. Photos: **A–C** by Kabir Montesinos; **D–G** by Álvaro J. Pérez.

**Figure 3.**
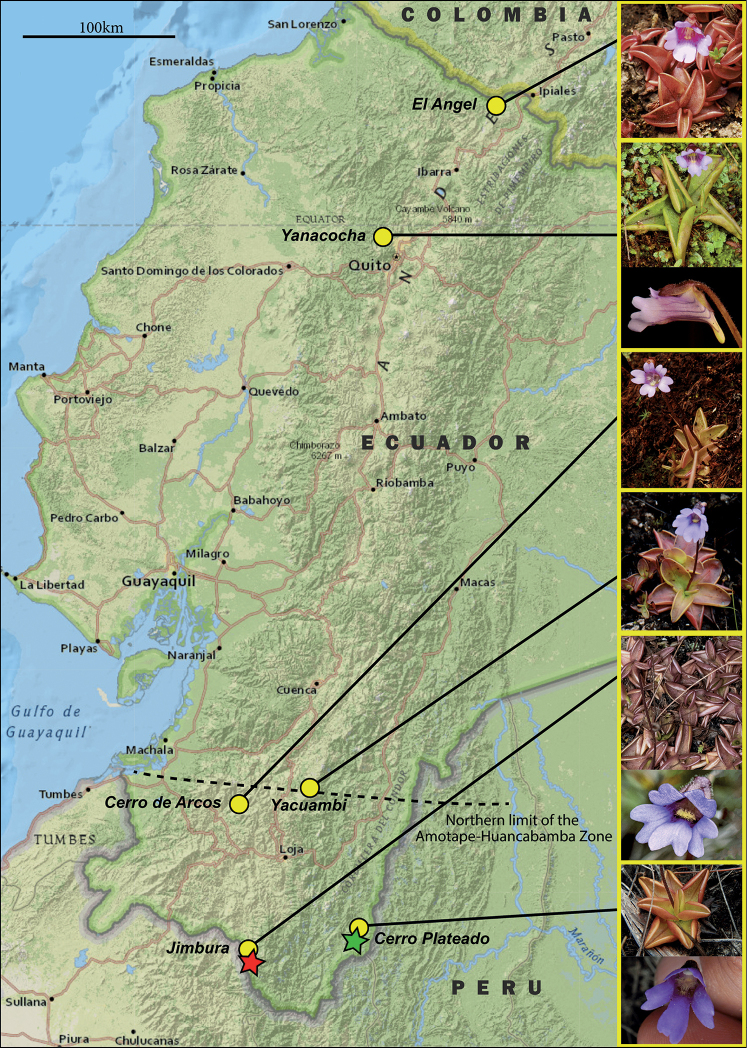
Map showing the distribution of *Pinguicula* spp. in Ecuador and illustrating the morphological spectrum of *P.calyptrata* observed from north to south. The yellow circles mark the respective localities sampled (locality name in italics). The two new species (*P.jimburensis* = red star, *P.ombrophila* = green star) are both found at the southern end of Ecuador near the border to Peru and well within the Amotape-Huancabamba Zone (dashed line).

Specimens of *P.jimburensis* have so far only been collected around the Lagunas Negras de Jimbura in the Yacuri National Park in the province of Loja. As a result, *P.jimburensis* is endemic and thus far only known growing between the grass and shrubby paramo vegetation around the lagoon complex, especially in swampy areas (Figs 2, 3). According to the Ministerio del Ambiente del Ecuador (2013), this locality lies within a much larger zone dominated by the ecosystem named arbustal siempreverde montano alto del Páramo del sur. Inhabitants of this area call this type of vegetation “paramillo” and the environment is characterized by a constant cloudiness and drizzle with strong winds. The vegetation is dominated by the herbs *Oritrophium* sp. *Paepalanthuslodiculoides* Moldenke, *Chusquea* spp. and *Phlegmariurus* spp., between the shrubby vegetation dominated by *Chuquiragajussieui* J.F. Gmel., *Monticaliaperuviana* (Pers.) C. Jeffrey and *Miconia* spp. Additionally, *Pinguiculacalyptrata* has been collected in this locality (*Pérez et al. 8690*, *8755*, QCA; Figs 2, 3). The border between Ecuador and Peru is only about 3 km in a straight line from the type locality, hence *P.jimburensis* could also occur in Peru.

##### Conservation status.

Only one population of approximately 50 mature individuals of this species was discovered at the type locality at the Yacuri National Park. The habitat is very close to the shore of the Laguna Negra and exposed to human activities related to spiritual rituals. Additionally, the trail that connects the lagoon complex closely passes by the population of this new species. According to the IUCN Red List criteria (IUCN Standards and Petitions Committee 2022) this species is assessed as Vulnerable (VU, Criterion D2).

##### Affinities.

*P.jimburensis* apparently is a close ally of *P.calyptrata* as are all Ecuadorian and Andean taxa. A similar atypical leaf orientation, with erect, elongated leaves can only be found in *P.elongata* Benj. from Colombia and Venezuela. This strange species is, contrary to traditional placements (Ernst 1961, Casper 1966), not closely related to the other Southern American taxa, but represents an isolated lineage with affinities to Mexican and European clades, as a recent molecular phylogenetic analysis has shown (Shimai et al. 2021). Moreover, the leaves of *P.jimburensis* are only slightly elongated and otherwise flat with a distinct abaxial and adaxial leaf lamina and not involute as the almost filiform leaves of *P.elongata*, with the abaxial leaf surface sometimes completely hidden. Furthermore, the leaf margins of *P.jimburensis* are irregularly, shallowly lobed, a unique character among *Pinguicula* from South America so far. With the lack of a yellow palate and the evenly narrowed spur that resembles the ripper tooth of a predatory mammal in shape, the flowers appear more similar to those of *P.involuta* than to typical flowers of *P.calyptrata*. The latter is characterized by the presence of a yellow palate and the spur, although very variable, usually has a stubby apex preceded by a narrower or at least straight section that is rather abruptly angled downwards after the throat. Both *P.jimburensis* and *P.involuta* lack a yellow palate and the thorn-shaped, regular curved spurs in both taxa are evenly narrowed towards the apex. However, *P.involuta* has a very different leaf morphology and is overall a much smaller taxon distributed much further south. *P.jimburensis* cannot be confused with any other American *Pinguicula* species based on the exceptional leaf morphology and orientation.

##### Additional specimens examined

**(*paratypes*).** Ecuador. Loja. Cantón Espíndola, Parroquia Jimbura, Parque Nacional Yacuri, Lagunas Negras de Jimbura, 04°42'S, 79°25'W, 3550 m, 10 Sept 2001 (fl), *P. Lozano & R. Bussmann 7* (LOJA, barcode: 31115); ibid, 04°42'46"S, 79°25'50"W, 3100–3200 m, 15 Oct 2018 (fl), *G. Salazar et al. 10191* (QCNE, barcode: 263036).

##### Photographic evidence.

https://www.inaturalist.org/observations/138176370.

#### ﻿ Pinguicula ombrophila

Taxon classificationPlantaeLamialesLentibulariaceae

Á.J.Pérez, Tobar & T.Henning sp. nov.

83551915-F373-502E-AFE9-DEB9048BB72D

urn:lsid:ipni.org:names:77316267-1

Fig. 4

##### Type.

Ecuador. Zamora-Chinchipe, Cantón Nangaritza, Parroquia Nuevo Paraíso, Reserva Biológica Cerro Plateado, −4.6194445, −78.7830556, 2850–2900 m, 27 Sep 2016, *Á.J. Pérez*, *N. Zapata & W. Santillán 10353* (holotype QCA (fl, fr) barcode: 245582).

##### Diagnosis.

*Pinguiculaombrophila* belongs to Pinguiculasect.Ampullipalatum and is closely allied to the other North Andean species of the section (*P.calyptrata*, *P.jimburensis* and *P.rosmarieae*). It differs from *P.calyptrata* and *P.jimburensis* in its lithophytic (vs. terrestrial) habit and the combination of broad rosettes with flat leaves (unlike *P.jimburensis*) without curled margins (unlike *P.calyptrata*). The flowers of *P.ombrophila* are similar to those of *P.calyptrata* and a distinct yellow palate – absent in *P.rosmarieae* and *P.jimburensis* – is present. It differs from all three other taxa by having very short flower scapes that barely reach leaf-length.

**Figure 4.**
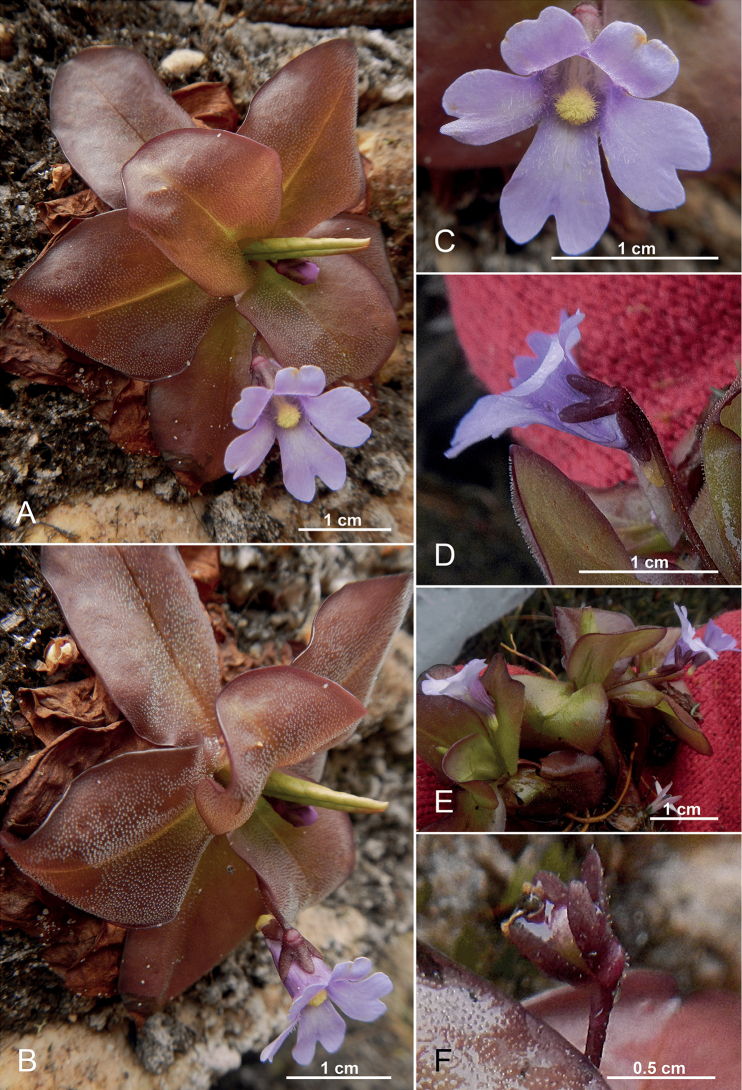
*Pinguiculaombrophila***A, B** flowering plant in fronto-lateral view in the natural habitat in the Reserva Biológica Cerro Plateado **C** flower, frontal view **D** ditto, lateral view **E** freshly collected specimen, note the angled spurs with the blunt apices **F** young capsule. (**A–F** from *Á.J. Pérez et al. 10353*). Photos by Álvaro J. Pérez.

##### Description.

Lithophyte on sandstone rocky walls, perennial rosette leaved herb with 1 (–3) flowering scapes. Rhizome ~12 mm long, with numerous fibrous roots 1.5–6.5 cm long. Leaves (5–) 6–7, flat on the ground, ± succulent (dried translucent-membranous), (15–) 20–30 (–35) mm long, nearly as long as wide, the blades ovate-obovate-oblong in outline, rounded at the tip, attenuated to the base into a sessile petiole, the margins entire, light green along the midvein and purple-brownish throughout the rest, upper surface of lamina covered with stalked glandular hairs. Hibernacula (winter buds, dormant buds) absent. Scapes 1–2 (–3), erect, (20–) 25 (–30) mm tall, terete, filiform (0.5–1 mm thick), one-flowered, purple-brownish, scattered with stalked glandular hairs. Flowers small, ~10–13 (–15) mm long (including tube-spur-complex). Calyx two-lipped, persistent, purple-brownish, upper surface of sepals scattered with stalked glandular hairs; upper lip deeply divided into three nearly equal-sized oblong lobes, at apex pointed; lower lip deeply divided into two oblong lobes, at apex pointed. Corolla two-lipped, bluish-magenta to bright-violet, scattered with stalked glandular hairs; upper lip two-lobed, lobes obovate, ~5–6 mm long and ~4–6 mm wide, notched at the apex; lower lip larger and longer than the upper lip, with three oblong to obovate-oblong lobes (the median lobe somewhat larger than the two lateral ones), 5–6 mm wide, each distinctly (up to 1/3 of its length) notched. Tube (tube-spur-complex) at the throat funnel-shaped and with dark stripes, proximally getting narrower until the end of the lower calyx lobes and merging relatively abruptly and with a weak angle into the cylindrical stubby, at apex rounded, yellow spur; ~6 mm long; Palate simple, well developed, inserted immediately behind (~1–2 mm) the corollas´ lower-lip middle lobe, yellow, set with short-stalked glandular hairs, proximally elongated into two short ventral hair strips; the corollas´ inner surface covered with small white hairs, stretching proximally along on each side of the inner tube wall. Stamens 2, filaments 1.5–2 mm long, anthers dorsifixed, 0.5 mm, oval, transverse dehiscing. Ovary 1.2 mm, rounded, glabrous, style 1 mm long, stigma 1 mm long, campanulate, sparsely covered with simple trichomes. Capsule 3–4 mm, rounded, glabrous, splitting in 2 valves. Seed numerous, alveolate, ellipsoid, 0.5–0.8 mm long. Chromosome number unknown.

##### Etymology.

The specific epithet was chosen to point at the particular habitat requirements of the plants. They prefer very wet conditions where they not only receive constant moisture from the surrounding waterlogged paramo-soil, but are also fully exposed to the high amounts of precipitation and fog typical for this area. The name ombrophila signifies “rainloving” from the two latin words “ombros” (rain) and “philos” (that loves/is fond of).

##### Distribution, habitat and associated vegetation.

The Cerro Plateado Biological Reserve is one of the governmental protected areas along the Cordillera del Cóndor range in Ecuador, protecting around 26 000 ha of mature forest from 850 to 3100 m in the province of Zamora-Chinchipe. The Cordillera del Cóndor runs 150 km north-south along the border of Ecuador and Peru. This mountain range is isolated from the main Andean chain and is geologically distinct, formed with an intermixture of limestone, quartzitic sandstone, and igneous rock of the Hollin Formation (Gregory-Wodzicki 2000; Neill 2005). The geology of these mountains is similar to the tepuis of the Guyana shield in northwest South America. In fact, a number of angiosperm genera once thought to be endemic to the tepuis of the Guyana shield have also been found along the Cordillera del Cóndor (Berry et al. 1995; Schulenberg and Awbrey 1997).

*Pinguiculaombrophila*, together with *P.calyptrata* (*Neill et al. 17467*, ECUAMZ; *Pérez et al. 10170*, *11711*, QCA; Figs 2, 3), were collected during the first botanical expedition to the Cerro Plateado led by Dr. David A. Neill in 2012 and later in 2016 and 2021 by the first author of this paper. Both species were collected at the summit of this Andean tepui, very close to the Peruvian border, that correspond to the highest sandstone plateau of the Cordillera del Cóndor, growing as a lithophyte on exposed quartzitic sandstone rocks with acidic and nutrient poor soils and in an extremely wet environment (Figs 2, 3). The vegetation is paramo-like, dominated by the grass Chusqueacf.nana and the bromeliad *Vriesea* sp. along with scattered shrubs of *Clethraconcordia* D.A.Neill, H.Beltrán & Quizhpe, *Diplostephium* sp., *Gaultherialanigera* Hook. and *Valerianaplateadensis* sp. nov. Á.J. Pérez, C. Persson & J.N. Zapata (Persson et al. 2023).

##### Conservation status.

Only one population with ca. 15 mature individuals of this species was discovered at the type locality at the summit of the Cerro Plateado. It is an isolated area and difficult to access; nevertheless, climate change could affect the environmental requirements of this species. According to the IUCN Red List criteria (IUCN Standards and Petitions Committee 2022) this species is assessed as Vulnerable (VU, Criterion D2).

##### Affinities.

*P.ombrophila* is superficially very similar to *P.rosmarieae* with which it shares the wide leaves without involute margins and the resulting large and relatively flat rosettes. Interestingly, both *P.ombrophila* and *P.rosmarieae* are lithophytic (the latter rarely epiphytic) plants that prefer the wettest of all non-submerse locations available in the generally very moist paramo habitats. The rosette morphology likely is an adaptation to that and might be subject to environmental constraints. *P.rosmarieae* shows, however, a different flower morphology with a peculiar box-like corolla-spur complex and the lack of a yellow palate. The flowers of *P.ombrophila* are more similar to *P.calyptrata* and the three species are obviously closely related to each other. *P.ombrophila* is furthermore characterized by a very short flower stalk compared to all other north-Andean taxa. The flowers barely exceed the tips of the freshly developing leaves, a character so far only known and typical for, for example, the south-Andean *P.nahuelbutensis* (Gluch 2017).

##### Additional specimens examined

**(*paratypes*).** Ecuador. Zamora-Chinchipe. Cantón Nangaritza, Reserva Biológica Cerro Plateado, Herbaceous páramo-like vegetation on broad, gently sloping summit area of Cerro Plateado, 04°37'10"S, 078°46'59"W, 2915 m, 24 Aug 2012 (fl), *D. Neill et al. 17465* (ECUAMZ); ibid, colecciones en la cima de la meseta, vegetación paramuna, −4.6194445, −78.7830556, 2900 m, 23 Sep 2016 (fl, fr), *Á.J. Pérez et al. 10145* (QCA, barcode: 245583); ibid, 8 Aug 2021 (fl, spirit collection), *Á.J. Pérez et al. 11712* (QCA, barcode: 245580).

## ﻿Discussion

The results presented in this study show that the assessment of the neotropical biodiversity is far from complete. Even in well-known groups such as the carnivorous plants, new taxa are continuously discovered and described, in particular from remote areas that become accessible in the course of the unlimited urban sprawl. This is both encouraging and worrying at the same time. While there are evidently still pristine habitats left that inhabit an unknown biodiversity, the fact that these ecosystems are now at an accessible distance to human infrastructure puts them under immediate threat of exploitation and destruction. In particular, the eastern slopes and Andean foothills facing Amazonia in the northern Andes border such remaining microhabitats in the form of isolated paramo sites and cloud forest fragments. Their seclusion in relation to similar neighboring habitats by the rugged terrain led to a mosaic of small scale organismal communities that are often self-contained to a certain extent. In the case of flowering plants, the degree of isolation often is determined by the associated pollen vector, usually the pollinating fauna. While spatial isolation of plant-pollinator communities is considered the main driver of diversification in angiosperms, intra-population competition for resources can also play an important role. In the case of insectivorous *Pinguicula* two potentially limited resources appear to be essential: pollinators and prey. Striving for these resources between neighboring populations can lead to rapid phenotypic diversifications in morphologically plastic parts of the plant body such as leaves and flowers. Furthermore, in co-occurring taxa, these changes can be strongly accentuated towards a differentiation by a phenomenon known as character displacement (Beans 2014). An adaptation for different prey by e.g. the different shape and orientation of the leaves in the sympatric taxon pairs found can only be assumed. However, the differences observed in the flower morphology between populations, both sympatric and throughout the entire distribution range of *P.calyptrata* s.l., point at effective reproductive barriers. The fact that, as far as is known, all species of the section (i.e. *Ampullipalatum*) can be artificially hybridized, but no hybrids have been reported from the wild (Fleischmann 2021) indicates that there are no genetic barriers, but other factors in effect. Plant-pollinator interactions can be very specific and are often controlled by multiple factors, especially in heterogeneous mosaic-like landscapes such as the high Andes. In addition to the morphological adaptations of flowers (e.g., color, size, spur length), traits such as nectar quantity and sugar concentration are often precisely matched to a particular pollinator taxon (Ackermann and Weigend 2006), and even pollen presentation can be the subject of complex and specific plant-pollinator interactions (Henning et al. 2018). Although speculative, such processes likely play a key role in the ongoing diversification of *Pinguicula* and evidently in the phenotypic divergence observed in the Ecuadorian taxa. Studies on the pollination biology of *Pinguicula* are scarce, the specificity of their pollination ecology is largely unknown, and appropriate research is strongly encouraged. However, published pollinator observations as e.g. in the recently described *P.warijia* from Mexico (Zamudio et al. 2023), provide evidence for a single principal pollinator (butterflies) and a respective close interaction.

### ﻿Morphological variation of *Pinguiculacalyptrata* Kunth

*Pinguiculacalyptrata* shows a great morphological variability in all phenological aspects throughout its distributional range and the different populations considerably vary in size, shape and color of all parts of the plant body (Figs 2, 3). Since the protologues of the “historical” taxa are usually based on a single collection and neither provide measurements, nor by any means outline a morphological spectrum, the real taxonomic extent of *Pinguicula* remains hidden in the broad taxonomic concept pursued ever since (Casper and Hellwig 2019; Casper et al. 2020). This is particularly true for *Pinguiculacalyptrata* and likely holds true for other South American taxa not yet studied in the necessary depth. The present description of the two new species should be understood as the continuation of an ongoing taxonomic extension of the genus as anticipated once a thorough molecular study including material from as many populations as possible can be conducted. Such a study has recently shed light on the taxonomy and nomenclature of the *P.vulgaris* aggregate in Europe, which, based on conflicting taxonomic concepts and different methodological approaches, were subject to scientific debates for decades (Majeský et al. 2022). The earlier separations of *P.jarmilae*, *P.nahuelbutensis* and *P.rosmarieae* from their putative sister taxa (*P.involuta*, *P.antarctica* & *P.calyptrata* respectively) were already founded on the evaluation of new collections and their morphological divergence from the broad circumscriptions of the historical taxa. In the case of *P.jarmilae*, which was later and hence invalidly also described as *P.chuquisacensis* by Beck et al. (2008), the latter authors already conducted a molecular study to underscore species delimitation and reveal the relationships among the South American taxa. Their key findings were that, contrary to their initial assumption, not morphologically more similar *P.calyptrata* but *P.involuta* is the closest relative and that the new species showed an unexpectedly high amount of autapomorphies in its sequence data (plastid markers). Furthermore, their preliminary dataset revealed a high genetic divergence among the South American taxa relative to those found in other clades of *Pinguicula*. These findings contrast with the comparatively low number of taxa described from Andean South America to this day. We therefore expect that the number of taxa will grow with the availability of molecular data and a concomitant better understanding of the taxon boundaries and speciation processes in this group of plants. For the time being, we must confine ourselves to describe those taxa as new to science, whose delimitation based on morphology and biogeography alone appears objectively evident.

The newly described species and the still unsatisfactorily documented and understood diversity among Andean *Pinguicula* underline the need to continue with botanical explorations and taxonomic studies and intensify urgently needed conservation efforts. The actual diversity of the Andean flora, in particular that of Ecuador and Northern Peru (AHZ), is still not conclusively determined. The threats to natural ecosystems in general, and those to small scale mountainous habitats in particular, are very worrying. *Pinguicula*, which inhabits very specific ecological niches due to its peculiar habitat requirements, is particularly susceptible to the destructive effects of e.g. mining activities and infrastructure projects (Cross et al. 2020), which penetrate further and further into the remaining remote pristine refugia. To make things worse, climate change increasingly affects local precipitation regimes and temperature profiles, with potentially dramatic consequences for moist habitats such as the paramo wetlands of the high Andes and their unique flora and fauna.

## Supplementary Material

XML Treatment for Pinguicula jimburensis

XML Treatment for Pinguicula ombrophila
